# Widespread land-use change may be caused by adaptation to climate-induced yield reductions

**DOI:** 10.1016/j.isci.2026.116973

**Published:** 2026-07-29

**Authors:** Bo Fu, Yongye Jiang, Xinhao Suo, Guolong Chen, Yuqin Lai, Chenxi Liu, Yuxin Huang, Bengang Li

**Affiliations:** 1 College of Urban and Environmental Sciences, Institute of Carbon Neutrality, Peking University, Beijing 100871, China; 2 Institute of Reproductive and Child Health, School of Public Health, Peking University, Beijing 100191, China; 3 School of Public Health, Sun Yat-sen University, Guangdong 518057, China

**Keywords:** climate change, agriculture, land use change, climate adaptation

## Abstract

Agriculture is at the center of the relationship between human societies and natural systems, acting as both a major source of greenhouse gas emissions and a sector highly sensitive to climate change. When climate change reduces agricultural yields, land-use change represents a key pathway through which human societies may adapt. Here, we coupled a climate-yield emulator with an integrated assessment model to quantify how adaptation to climate-induced yield losses reshapes the global land-use patterns. We found that adaptive responses to declining yields could substantially transform global land use. Under the Shared Socioeconomic Pathway 2-4.5 (SSP2-4.5) scenario, global cropland expands by approximately 1.2 million km^2^ (about 8%), largely at the expense of forests, grasslands, and pastures. This expansion is nearly five times greater under the SSP5-unconstrained scenario and is accompanied by additional warming. Even when endogenous integrated assessment model (IAM)-based adaptations are combined with additional enhanced management practices and technological advancements, land-use change remains a plausible response to compensating for yield declines. These results reveal an underappreciated risk associated with agricultural adaptation to climate change and underscore the importance of limiting temperature rise to safeguard food security while avoiding large-scale conversion of natural land.

## Highlights

• Climate-induced yield losses drive cropland expansion as adaptation
• SSP2-4.5 leads to 1.2 million km^2^ of additional cropland
• High warming amplifies land-use change and causes extra warming
• Management and technology reduce but do not eliminate land pressure

## Graphical abstract

**Figure:**
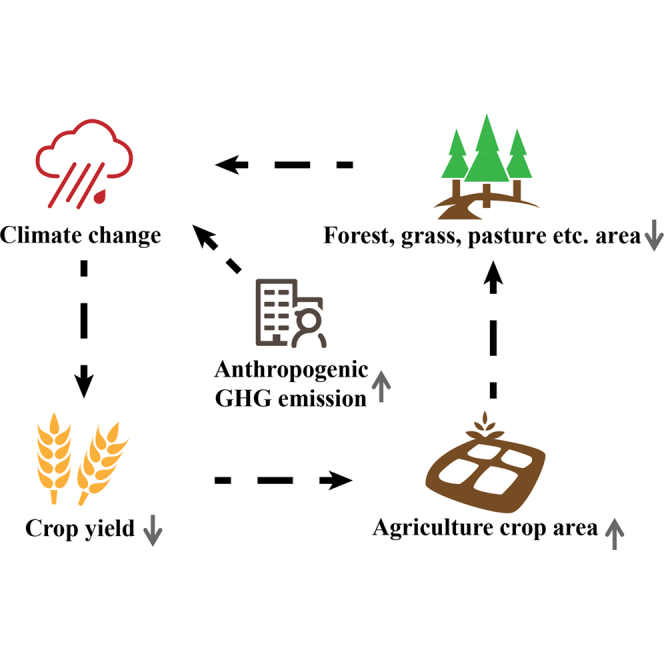

## Teaser

Environmental science; Applied sciences

## Introduction

Food security is increasingly being threatened by climate change.^1^^,^^2^^,^^3^ Rising temperatures and changing precipitation patterns affect crop production worldwide,^4^^,^^5^ while climate change also disrupts the storage, processing, distribution, and exchange of food, placing additional stress on food systems. These risks are likely to intensify because the world’s population is projected to continue growing through 2050 in most Shared Socioeconomic Pathway (SSP) scenarios.^6^ In response to these escalating challenges, the World Food Program (WFP) has called on the global community to prevent climate change from contributing to a 20% increase in the number of hungry people by mid-century.^7^

A range of mitigation and adaptation measures have been proposed to reduce climate-related risks to agricultural production, including improving crop varieties, enhancing agricultural management, reducing food loss and waste, and expanding cropland.^8^^,^^9^ Among these options, cropland expansion is particularly concerning because it poses substantial threats to biodiversity, ecosystem integrity, and environmental sustainability.^10^^,^^11^^,^^12^ Nevertheless, global cropland expansion has continued over recent decades despite sustained yield improvements and rising agricultural productivity,^13^^,^^14^ and recent evidence suggests that a substantial share of this expansion can be attributed to climate change.^15^ From a food security perspective, recent work further indicates that maintaining sufficient food supply under a changing climate may require either cropland expansion or substantially strengthened alternative adaptation measures.^16^

This process may generate reinforcing feedback between climate change, agricultural production, and land use. Climate change can reduce agricultural productivity, thereby increasing pressure to convert additional land to agricultural land, and cropland expansion can, in turn, reinforce climate change through land-use-related greenhouse gas emissions and reduced natural carbon sinks.^17^ Historical observations already indicate the existence of such a climate-cropland feedback,^15^ but its potential magnitude under future warming scenarios remains uncertain. Moreover, although previous studies have examined climate impacts on crop yields, land-use adaptation, and agricultural mitigation pathways,^18^^,^^19^ most assessments have treated the sequence from climate change to yield change and land-use response as largely a one-way process. The extent to which adaptation-driven land-use change may feedback onto the climate system and whether enhanced management practices or technological progress can constrain this feedback has not yet been systematically quantified. Addressing these questions requires a modeling framework that explicitly couples climate change, agricultural productivity, land-use decisions, land-use-related emissions, and the resulting climate feedbacks.^19^

In this study, we couple a climate-yield emulator^2^ with the Global Change Analysis Model (GCAM) v.7.0^20^ to represent two-way interactions among climate change, crop yields, land use, and land-use-related emissions. This coupled framework differs from conventional one-way modeling approaches in that it allows climate-induced yield changes to alter agricultural production and land allocation, while the resulting land-use changes can further affect emissions and climate. We quantify the contribution of these human-nature interactions by comparing coupled simulations with uncoupled GCAM reference simulations. Specifically, we simulate yields, production, and harvested area for fifteen crops, including three staple crops, across three SSP-Representative Concentration Pathway (RCP) scenarios under different assumptions (Table 1). We then assess how climate-induced yield losses and human adaptation reshape global land-use patterns, alter regional food self-sufficiency, and generate cascading effects across the climate-food-land system. Finally, building on previous studies,^21^^,^^22^ we conduct sensitivity tests to evaluate whether enhanced agricultural management and optimistic technological advancements can mitigate climate-driven cropland expansion.

**Table 1:** Scenarios simulated in this study

SSP	RCP	Climate-yield impacts	Enhanced management practices	Technological advancements
SSP1	2.6	no	not applied	default as GCAM
SSP1	2.6	yes	not applied	default as GCAM
SSP1	2.6	yes	applied	default as GCAM
SSP1	2.6	yes	not applied	addition 1/3 APG^a^
SSP2	4.5	no	not applied	default as GCAM
SSP2	4.5	yes	not applied	default as GCAM
SSP2	4.5	yes	applied	default as GCAM
SSP2	4.5	yes	not applied	addition 1/3 APG^a^
SSP5	no target	no	not applied	default as GCAM
SSP5	no target	yes	not applied	default as GCAM
SSP5	no target	yes	applied	default as GCAM
SSP5	no target	yes	not applied	addition 1/3 APG^a^

a Short for the agricultural productivity growth rate, representing future technological advancements.

## Results

### Yield reduction induced by climate change

**Figure 1:**
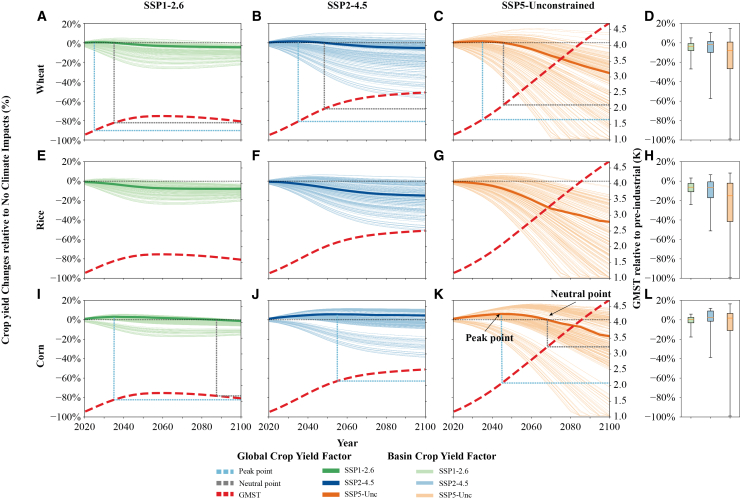
The climate impact on three staple crops in the SSP-RCP scenarios (A–C, E–G, and I–K) Relative yield changes for wheat (A–C), rice (E–G), and maize (I–K) under SSP1-2.6, SSP2-4.5, and SSP5-unconstrained scenarios. Thick lines indicate changes in global mean yields relative to simulations without climate-yield impacts, and thin lines indicate basin-level yield changes across 235 GCAM hydrologic basins. Red lines show the increase in global mean surface temperature (GMST) relative to pre-industrial levels. Dashed blue and gray lines mark the peak and neutrality points of global mean yield changes where they occur. (D, H, and L) Boxplots summarizing the distribution of basin-level yield changes across scenarios; center lines indicate medians, boxes indicate interquartile ranges, and whiskers indicate the full range from the minimum to maximum values.

Although atmospheric CO_2_ concentrations are well mixed globally, climate change produces spatially heterogeneous warming, leading to regionally differentiated impacts on crop yields. Here, global mean surface temperature (GMST) refers to the increase in global mean surface temperature relative to pre-industrial levels. In our coupled simulations, GMST trajectories are downscaled to basin-level growing-season temperatures and combined with atmospheric CO_2_ concentrations to estimate climate-induced yield changes across the 235 hydrologic basins represented in GCAM. Figure 1 shows relative yield changes for three major staple crops—wheat, rice, and maize—under SSP1-2.6, SSP2-4.5, and SSP5–unconstrained scenarios. Thick lines indicate changes in global mean yields, whereas thin lines show basin-level yield responses. The dispersion of basin-level trajectories indicates that climate impacts on crop yields are highly heterogeneous across regions, even when global mean yield changes appear relatively smooth. Corresponding results for other crops are shown in Figures S1–S3.

The responses of the three staple crops differ substantially across scenarios and crop types. Wheat yields show a limited initial increase, followed by declines, under all scenarios. Under SSP1-2.6, global mean wheat yield changes remain small throughout the century, ranging from +0.6% to −4.5%. Under SSP2-4.5, wheat yield changes are similar in magnitude, ranging from +1.5% to −5.3%, because stronger CO_2_ fertilization partly offsets the effects of higher warming. However, regional losses become more pronounced, with wheat yield reductions exceeding 20% in some basins. Under SSP5–unconstrained scenario, continuous warming leads to a much stronger decline, with global mean wheat yield reductions reaching 31.1% by the end of the century. Rice is more adversely affected than wheat or maize. Global mean rice yields decline throughout the century in all three scenarios, with end-of-century losses of 7.9%, 14.8%, and 41.9% under SSP1-2.6, SSP2-4.5, and SSP5–unconstrained, respectively. Regional impacts are substantially larger than global mean changes, reaching approximately 20% in the most affected regions under SSP1-2.6 and approximately 50% under SSP2-4.5. Under SSP5-unconstrained scenario, some regions experience rice crop failure after 2075. Maize shows a different response because its optimal growing temperature is higher than that of wheat and rice. Climate-induced changes in global mean maize yield remain close to neutral under SSP1-2.6, ranging from −1.2% to +3.1% and become positive under SSP2-4.5, with a global increase of 5.7%. However, this positive response does not persist under high warming: under SSP5-unconstrained scenario, the climate effect on maize yield shifts from positive to negative around 2070, after which continued warming reduces yields.

Overall, these results show that warming mitigation substantially reduces climate-induced yield losses and prevents the emergence of severe regional crop failures. Low- and intermediate-warming pathways produce broadly similar global wheat and maize responses, but SSP1-2.6 provides a clear advantage for rice, which is the crop most sensitive to warming in our simulations. By contrast, SSP5-unconstrained leads to large yield reductions across all three staple crops and generates regional crop failures after 2070. These losses imply stronger dependence on interregional food supply and greater pressure on land-use systems, providing the basis for the land-use responses analyzed in the following section.

### Land-use responses to climate-induced yield reductions

**Figure 2:**
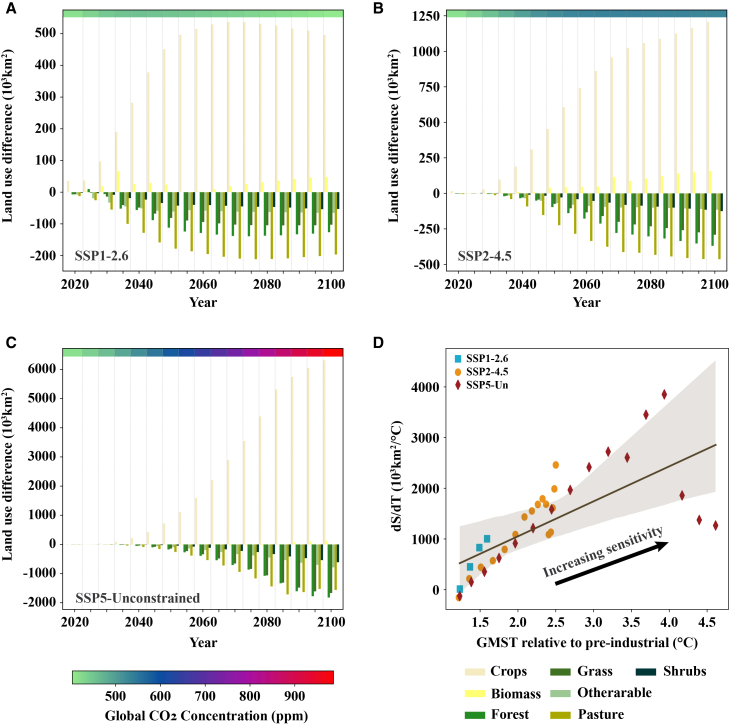
Climate-driven land-use change and sensitivity of cropland expansion to warming (A–C) Bar plots showing land-use changes induced by climate-yield impacts under SSP1-2.6, SSP2-4.5, and SSP5-unconstrained scenarios. Positive values indicate land expansion, and negative values indicate land contraction relative to uncoupled simulations without climate-yield impacts. (D) Sensitivity of cropland area to warming, calculated as dS/dT, where S is cropland area and T is GMST. Only years in which GMST and CO_2_ concentration both increase are used to estimate the sensitivity. The fitted line shows the regression relationship between warming and cropland sensitivity, and the shaded band indicates the 95% confidence interval of the fitted regression line.

Climate-induced yield reductions reshape global land-use patterns through adaptive responses in the agricultural system, most notably through cropland expansion. We, therefore, quantify land-use changes induced by climate-yield impacts across SSP1-2.6, SSP2-4.5, and SSP5-unconstrained scenarios (Figure 2). Across all scenarios, cropland expansion is the dominant land-use response, but its magnitude differs sharply with the level of warming. Under SSP1-2.6, climate-driven cropland expansion peaks at approximately 540,000 km^2^ around 2070, following the trajectory of moderate warming and subsequent stabilization. Under SSP2-4.5, continued warming leads to an end-of-century cropland increase of approximately 1,207,000 km^2^, more than double the peak expansion under SSP1-2.6. Under SSP5-unconstrained scenario, much stronger warming drives a far larger cropland expansion, reaching approximately 6,320,000 km^2^ by the end of the century, nearly five times the increase under SSP2-4.5.

**Figure 3:**
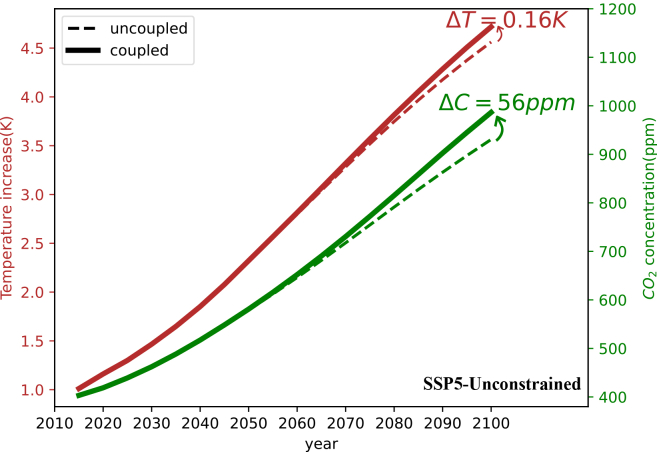
Additional warming caused by adaptation-driven land-use feedback under SSP5-unconstrained Solid lines show coupled simulations in which climate-induced yield changes affect land use and land-use-related emissions. Dotted lines show uncoupled reference simulations. Red lines indicate GMST increase relative to pre-industrial levels, and green lines indicate atmospheric CO_2_ concentration.

The divergence in cropland expansion across scenarios reflects the nonlinear relationship between warming, crop yield losses, and land demand. In SSP1-2.6 and SSP2-4.5 scenarios, cropland expansion remains below 1% of global land area, whereas under SSP5-unconstrained scenario, it reaches approximately 5% of the global land area. This large-scale expansion further affects the climate system by increasing land-use-related CO_2_ emissions. In SSP5-unconstrained scenario, the additional emissions generated by adaptation-driven land-use change contribute approximately 0.16°C of extra warming by the end of the century (Figure 3). By contrast, no significant additional warming is detected under SSP1-2.6 or SSP2-4.5 scenario. These results indicate that the land-use feedback is weak under lower-warming pathways but becomes detectable under high warming, where yield losses are larger and cropland expansion is more extensive.

The sensitivity of cropland area to warming, defined as the increase in cropland area per unit of GMST increase, also rises with temperature (Figure 2D). At lower levels of warming, the cropland response is relatively limited, with sensitivity on the order of hundreds of thousand km^2^ per degree of warming. Under stronger warming, sensitivity increases to thousands of thousand km^2^ per degree, indicating that cropland demand accelerates as climate-induced yield losses become more severe. This nonlinear response arises from nonlinear crop-yield responses to temperature and the saturation of the CO_2_ fertilization effect. At warming levels above approximately 4°C, however, cropland sensitivity begins to decline because some low-yield regions no longer expand cropland locally; instead, production shifts toward regions with more favorable yields, increasing reliance on interregional supply.

Land converted to cropland comes mainly from pastures, forests, and grasslands, but the relative contribution of these land types varies by scenario. Under SSP1-2.6 and SSP2-4.5 scenarios, pasture accounts for the largest share of cropland expansion, followed by forests and grasslands. Bioenergy land also increases by approximately 48,000 km^2^ under SSP1-2.6 and 157,000 km^2^ under SSP2-4.5. Under SSP5-unconstrained scenario, the scale of land conversion is much larger, with forests, grasslands, and pastures declining by approximately 1,821,000, 1,666,000, and 1,561,000 km^2^, respectively. Together, these results show that adaptation to climate-induced yield reductions can create substantial pressure on natural and semi-natural land systems, especially under high-emission pathways.

### Regional differences in cropland expansion, production, and food self-sufficiency

**Figure 4:**
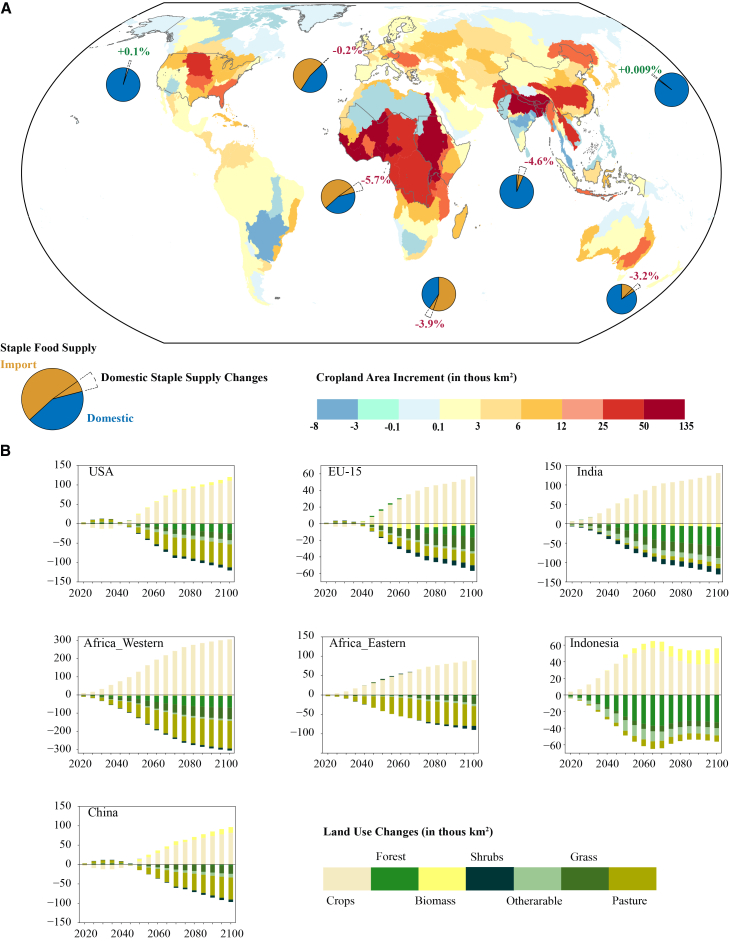
Regional patterns of cropland expansion and food self-sufficiency under SSP2-4.5 (A) Spatial pattern of cropland expansion at the end of the century induced by climate-yield impacts under SSP2-4.5. Seven representative regions are highlighted: the US, the EU-15, India, China, West Africa, East Africa, and Indonesia. Pie charts show the shares of domestic staple-food supply and imports in these regions, with annotations indicating changes in the self-sufficiency ratios. (B) Time series of land-use change in the seven representative regions, showing the sources and trajectories of cropland expansion and contraction over time.

SSP2-4.5 represents an intermediate development pathway and is broadly consistent with current global trends. We, therefore, use this scenario as an illustrative case to examine regional differences in land-use change and food-system responses. Relative to the uncoupled simulation, the coupled SSP2-4.5 scenario produces an additional 1,207,000 km^2^ of global cropland expansion by the end of the century. This increase is concentrated in Africa and Asia, which account for 470,000 and 380,000 km^2^ of additional cropland, respectively, corresponding to 38.9% and 31.6% of the global total (Figure 4A). Smaller increases are also found in North America, Europe, and Oceania. These results indicate that the global cropland response to climate-induced yield reductions is spatially uneven, with Africa and Asia emerging as the primary hotspots of land-use pressure.

Across the 32 geopolitical regions represented in GCAM, the consequences of cropland expansion differ markedly. West Africa shows the largest increase in cropland area, yet the production of the three staple crops still declines, and dependence on imported staple foods rises by 5.7% (Table S1). India and Indonesia show a similar pattern: cropland expands by 130,000 and 38,000 km^2^, respectively, but staple-food production declines and the self-sufficiency ratios decrease by 4.6% and 3.2%, respectively. Here, the self-sufficiency ratio refers to the proportion of food consumption that is met by production within the same region. By contrast, the US, the EU-15, and China also experience cropland expansion, but their staple food self-sufficiency remains nearly unchanged (Figure 4A; Table S1). More broadly, low-income regions tend to experience declining staple food self-sufficiency, whereas changes are much smaller in higher-income regions (Figure S4). The temporal trajectories of land-use change also vary across regions (Figure 4B). Cropland continues to expand in West Africa and India, whereas in the US, the EU-15, and China, it first declines and then increases. This contrast reflects the latitudinal pattern of climate impacts on crop yields: warming generally reduces yields in low-latitude regions but may initially benefit some middle- and high-latitude regions before becoming detrimental under stronger warming. By the end of the century, yield losses in some low-latitude regions, such as West Africa, East Africa, India, and Indonesia, become large enough that local production becomes less competitive, slowing or reversing cropland expansion and increasing reliance on food imports.

**Figure 5:**
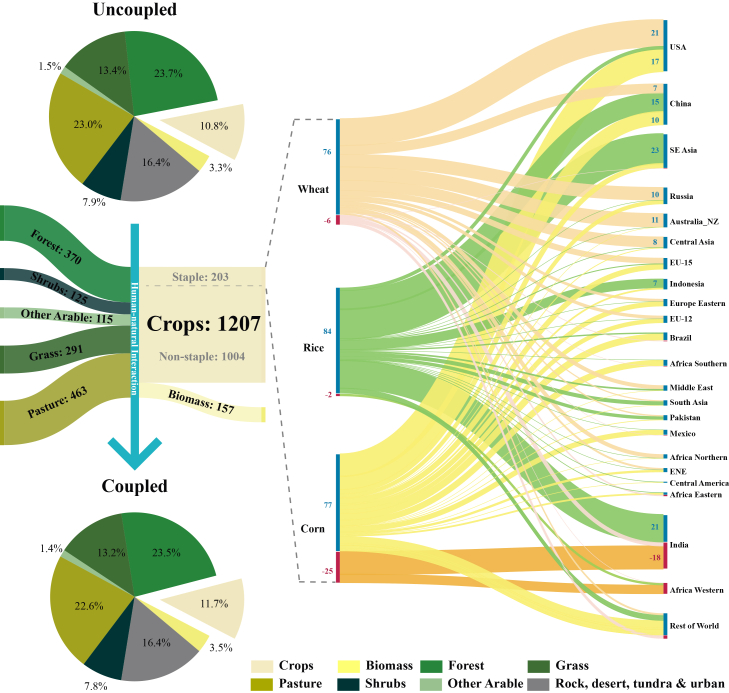
Global land-use reallocation and regional distribution of staple-crop expansion under SSP2–4.5 Left: comparison of the shares of major land-use types at the end of the century between uncoupled and coupled simulations, illustrating the expansion of cropland and the corresponding contraction of pastures, forests, and grasslands. The corresponding land-use shares under SSP1-2.6 and SSP5-unconstrained in the uncoupled simulations are provided in Figure S5. Right: the regional distribution of changes in rice, wheat, and maize areas, highlighting where expansion and contraction of the staple crop area occur.

Figure 5 summarizes how these regional responses translate into broader shifts in the land-use composition and staple crop allocation under SSP2-4.5. The global share of cropland increases from 10.8% to 11.7% of the total land area, corresponding to an 8% increase relative to the initial cropland area. This expansion comes mainly at the expense of pastures (463,000 km^2^), forests (370,000 km^2^), and grasslands (291,000 km^2^). The three staple crops together account for approximately one-fifth of the 1,207,000 km^2^ cropland increase, including 84,000 km^2^ for rice, 77,000 km^2^ for maize, and 75,000 km^2^ for wheat (Figure 5). The expansion of rice area is concentrated in Asia, with increases of 21,000 km^2^ in India, 15,000 km^2^ in China, and 7,000 km^2^ in Indonesia; together, these three countries account for more than a half of the global increase in rice area. Wheat area increases most strongly in the US (21,000 km^2^), followed by Australia, Russia, and China, each with increases of 7,000–11,000 km^2^. Maize expansion is also led by the US (17,000 km^2^), followed by China (10,000 km^2^), Brazil, Mexico, and Russia. Notably, the largest combined increases in staple crop area occur in the US and China, at 38,000 and 32,000 km^2^, respectively, even though these regions do not experience the largest increases in the total cropland area (Figure 4; Table S1). This pattern helps explain why staple food self-sufficiency remains relatively stable in the US and China but declines in many African regions.

## Discussion

Our findings show that climate-induced crop yield reductions can reshape global land-use patterns through human adaptation and, in turn, amplify climate warming through land-use-related greenhouse gas emissions. In response to declining yields, agricultural systems adjust production and land allocations, including through cropland expansion. Although such responses may partially buffer the food supply pressures, they also increase pressure on forests, grasslands, and pastures, while generating additional emissions that can reinforce warming. This feedback is weak under lower-warming pathways but becomes pronounced under SSP5-unconstrained, where cropland expansion is substantially larger and produces detectable additional warming. These results indicate that climate mitigation reduces land-use pressure not only by limiting direct warming but also by weakening the adaptation-driven feedback between yield losses, cropland expansion, and emissions.

This study extends previous work by explicitly representing two-way interactions among climate change, crop yields, land-use decisions, and land-use-related emissions. Earlier studies have quantified climate impacts on crop yields, land-use adaptation, or agricultural mitigation pathways, but many have treated the pathway from climate change to yield losses and land-use responses as a largely one-way process. By coupling a climate-yield emulator with the GCAM, we quantify how adaptation to yield losses feeds back onto the climate system through land-use change. This framework also allows us to distinguish between local cropland expansion driven by regional yield losses and broader cropland shifts driven by market-mediated redistribution of production across regions. As a result, our analysis shows not only where cropland expands but also how regional production, trade dependence, and staple food self-sufficiency change under climate-induced yield stress.

Within this framework, land-use adjustment is treated as an endogenous adaptation pathway, including cropland expansion, changes in crop allocation, and shifts in regional production. Other adaptation strategies may also reduce climate-induced yield losses, particularly improved agricultural management, irrigation management, fertilizer management, crop rotation, altered planting dates, and the development and deployment of improved crop varieties.^9^^,^^21^^,^^23^ To examine whether such measures could reduce pressure on land conversion, we conducted additional sensitivity experiments using published estimates of yield-loss mitigation from enhanced management practices.^21^ We also tested an optimistic technological scenario by increasing the default annual agricultural productivity growth rate in GCAM by one-third. These experiments show that enhanced management and accelerated productivity growth reduce but do not eliminate cropland expansion under substantial warming (Figure S6). Therefore, agricultural adaptation and technological progress are necessary but insufficient substitutes for climate mitigation, especially under high-emission pathways.

Overall, our results suggest that climate-induced yield reductions have implications not only for agricultural production but also for land conservation, food self-sufficiency, and welfare equity. The positive feedback in the climate-food-land nexus arises from interactions between humans and natural systems: as yields decline, societies adapt by expanding or reallocating cropland, and these land-use responses can further affect the climate system. Such expansion encroaches on forests, grasslands, and pastures, while potentially intensifying competition between food crops and bioenergy crops. Developing regions are likely to face greater challenges because they experience stronger land-use pressure and larger declines in staple food self-sufficiency. These findings highlight the need to evaluate mitigation costs, adaptation costs, and climate damages within a unified food security framework. If warming is limited in line with ambitious climate targets, the resulting food and land-use pressures are substantially reduced. If mitigation remains insufficient, however, adaptation to climate-induced yield declines may require land-use changes that place severe pressure on natural ecosystems and global food security systems.

### Limitations of the study

Several limitations should be noted. Climate change affects crop yields through multiple pathways, including warming,^4^^,^^24^ changes in precipitation patterns,^25^ elevated CO_2_ concentrations, and changes in the frequency and intensity of extreme weather events.^26^^,^^27^^,^^28^ It may also affect yields indirectly through pest and disease outbreaks^29^^,^^30^ and air pollution.^31^ However, sufficiently broad observational and experimental evidence is not yet available to robustly parameterize all of these mechanisms in global long-term simulations. In addition, the reduced-form climate module coupled within GCAM cannot simulate spatially explicit precipitation patterns or extreme events. We, therefore, consider only the effects of temperature and CO_2_ concentration on crop yields. Because several omitted mechanisms, such as extreme heat, drought, pests, and air pollution, are likely to further reduce yields in many regions, our estimates may be conservative with respect to future yield losses and the resulting land-use pressure. Further observational, experimental, and modeling work is needed to improve climate-yield relationships and to support land-use decisions that jointly address food security and climate mitigation.

## Resource availability

### Lead contact

Requests for further information and model should be directed to and will be fulfilled by the lead contact, Bengang Li (libengang@pku.edu.cn).

### Materials availability

This study did not generate new unique materials.

### Data and code availability

• GCAM v.7.0 is available at https://github.com/JGCRI/gcam-core.
• GCAM shapefiles are available from Zenodo: https://doi.org/10.5281/zenodo.4014308.
• The climate-yield emulator is available at https://github.com/rongwang-fudan/OSCAR_Agriculture_Global.
• The processed model outputs, figure source data, and plotting scripts used to generate the main and supplemental figures in this study have been deposited in Zenodo and are available at Zenodo: https://doi.org/10.5281/zenodo.20454811.

## Acknowledgments

This study is supported by the 10.13039/501100001809National Natural Science Foundation of China (grant no. 42521004) and the 10.13039/501100012166National Key R&D Program of China (no. 2022YFF0802503). We acknowledge Yang Ou and the GCAM community for their technical support on GitHub. Numerical computations were performed on Kunshan advanced computing center.

## Author contributions

Conceptualization, B.L. and B.F.; methodology, B.F., Y.J., and B.L.; software, B.F., Y.J., and Y.L.; investigation, B.F. and Y.J.; data curation, Y.J., X.S., and G.C., with support from C.L. and Y.H.; visualization, Y.J., X.S., and G.C.; writing – original draft, B.F., Y.J., and B.L.; writing – review & editing, all authors.

## Declaration of interests

The authors declare no competing interests.

## STAR★Methods

### Key resources table

**Table:** 

REAGENT or RESOURCE	SOURCE	IDENTIFIER
**Deposited data**		

Processed model outputs, figure source data, and plotting scripts generated in this study	This paper	Zenodo: https://doi.org/10.5281/zenodo.20454811
GCAM regional and basin shapefiles	GCAM community	Zenodo: https://doi.org/10.5281/zenodo.4014308
Climate–yield emulator and associated yield-response functions	Xu et al.^2^	GitHub: https://github.com/rongwang-fudan/OSCAR_Agriculture_Global

**Software and algorithms**		

GCAM v7.0 model input data and SSP–RCP scenario settings	PNNL Joint Global Change Research Institute/GCAM community	https://github.com/JGCRI/gcam-core

### Experimental model and study participant details

This study does not involve experimental models, biological samples, human participants, animals, cell lines, or clinical data. All analyses are based on numerical model simulations using GCAM v7.0, the Hector reduced-form climate module, and a published climate–yield emulator.

### Method details

#### Model framework and scenario design

To assess the two-way interactions between climate change, crop yields, and land-use dynamics, we couple the Global Change Analysis Model (GCAM)^20^ with a climate–yield emulator previously evaluated in a Nature paper.^2^ This coupled framework enables a consistent representation of how climate-induced yield changes affect agricultural production decisions, land allocation, greenhouse gas emissions, and the resulting climate feedbacks.

Within this framework, changes in temperature alter crop yields through the climate–yield emulator, which in turn influences land-use decisions in GCAM via adaptive responses aimed at maintaining agricultural output. These land-use adjustments affect terrestrial carbon stocks and anthropogenic emissions, leading to additional radiative forcing and warming that feeds back into the climate system. Our analysis focuses on this positive feedback loop linking yield losses, adaptive land-use change, carbon emissions, and additional warming, allowing us to quantify the extent to which climate adaptation in the agricultural sector may amplify pressures on natural land systems.

#### Global change analysis model

The Global Change Analysis Model (GCAM) v7.0, a community, open-source integrated assessment model (IAM) developed by the Joint Global Change Research Institute at the Pacific Northwest National Laboratory, is used to simulate future socioeconomic interactions with climate-induced yield change in this study. A detailed introduction to the GCAM is provided in ref.^20^ The GCAM v7.0 represents five systems, i.e., the economy, energy, agriculture and land use, water, and climate, spanning 32 geopolitical regions worldwide. It captures the interplay between land allocation, water usage, and agricultural production across 235 water basins. Within a partial equilibrium economic modelling framework, the GCAM simulates the dynamics and interplay among the five systems, projecting future patterns at five-year intervals under exogenous socioeconomic assumptions in each geopolitical region. The model utilizes a recursive dynamic methodology for cost optimization, determining the most cost-efficient energy configuration within the limitations of documented technological predilections for each period. Upon establishing this minimumost energy solution for a specified duration, it proceeds to the succeeding interval to replicate this optimization procedure. Therefore, every system will make choices that maximize its current interests.

In this study, different Shared Socioeconomic Pathways (SSPs) scenarios employ the corresponding SSP-RCP settings provided by the official GCAM, incorporating a Climate-yield coupling mechanism on this basis. Future agricultural activities are driven by the socioeconomic parameters (GDP, population, food demand, etc.) outlined in the SSP-RCP settings. The data preceding GCAM's final calibration year of 2015 is calibrated according to the FAO. The default agricultural productivity growth (APG) in GCAM is constructed based on the FAO's projected yields. Actions towards agricultural production reduction or increase under different SSP-RCPs vary, as do the rates of agricultural productivity growth. Based on a cost-optimization basis, within GCAM, a certain region may opt to utilize agricultural land to cultivate a crop commanding a higher price, re-cultivate fallow land, or convert other types of natural land use, such as forests or grasslands, into farmland. In this work, the increase in the area of a particular crop in a region is not solely due to adapting to climate change impacts on yields but also due to the economic equilibrium impacts of regionally differentiated impacts on yields. That is, regions with higher yields may choose to cultivate more land for crops to fill the demand gap caused by reduced production in other areas for profit. In GCAM, the main costs of agricultural planting include land prices, fertilizer prices, irrigation costs (surface water, groundwater, desalination), and carbon pricing, all of which are endogenous to GCAM and have regional characteristics. For instance, irrigation costs in arid regions will be higher than those in regions with abundant water resources. Therefore, if a particular crop in a GCAM region has too low a yield per unit area or the planting costs are too high, the choice may be made to cultivate less or not at all of this crop. Other regions may opt to cultivate more to fill this demand gap, thus leading to a Global Cropland Shift phenomenon.

Food demand also influences agricultural activities, where dietary preferences determine the amount of land used for crop production. In GCAM, future food demand in different regions changes with income and food prices, based on concepts proposed by Edmonds and others. As income increases, both total food demand and non-staple food demand correspondingly rise.^32^

The climate module in the GCAM is the open source reduced-form carbon-cycle climate module Hector v3.0. Hector can effectively reproduce past patterns and project future trends in atmospheric CO2 levels, radiative forcing, and global mean temperature changes. The GCAM can feed emission data to Hector to simulate climate change, while Hector has no feedback to the GCAM. Therefore, the original GCAM is unable to model the impacts of climate change on socioeconomic systems (e.g., declining crop yields). To date, there is limited literature attempting to incorporate Hector's climate data as feedback in driving GCAM, with only one known study^33^ focusing on building energy consumption. In this study, we couple a climate-crop emulator^2^ and a temperature downscaling module with the GCAM v7.0,^20^ making it possible to estimate the interaction between human and natural systems in agricultural activities.

#### Temperature downscaling module

Local surface temperatures in production areas are necessary to simulate the impact of temperature on agricultural yield per acre. However, Hector can calculate only the global mean surface temperature (GMST). To simulate the variations in agricultural yield across 235 basins under the influence of temperature, we establish a temperature downscaling module using the following formula to scale down the GMST to the local mean growing season temperature (LMGST):

(Equation 1) $$LMGST_{\left(Basin,t\right)}=LMGST_{\left(Region,2015\right)}+\left(GMST_{t}-GMST_{2015}\right)\times \gamma_{Basin}$$

*γ*_*Basin*_ represents the slope derived from establishing a linear correlation between the local mean surface temperature of each basin and the GMST using various historical and future global temperature datasets from ERA5 and CMIP6.

#### Climate-yield emulator

There are many pathways through which climate change may affect food yield, including increased temperature, changes in precipitation patterns, increased CO2 concentrations, and increased frequency and intensity of extreme weather events. However, in this study, we consider only the climate yield induced by temperature and CO2 due to the capabilities of Hector, a simple climate model, which is unable to replicate weather patterns and extreme weather events. Moreover, a substantial portion of agricultural crops in the GCAM rely on irrigation rather than rainfed practices.

In the GCAM, there are a total of eight agricultural crops (corn, fodder, miscellaneous crops, oil crops, other grains, root tuber, sugar crop, and wheat). We update the yields of all agricultural crops in each GCAM period, taking into account the influence of climatic conditions, using the climate-yield model proposed by Xu et al.^2^ Each crop’s climate-yield equation is estimated through field-warming experiments and local process-based or statistical models. Following Xu et al.^2^ yield-climate functions are developed for wheat, corn, and rice, and other crops are assumed similar to that for wheat.

In the model, the formula of agricultural yield calculation is as follows:

(Equation 2) $$yield_{t}=yield_{t-1}\times \left(1+APG\right)\times \frac{F^{T}\left(T_{t}\right)}{F^{T}\left(T_{0}\right)}\times \frac{F^{C}\left(C_{t}\right)}{F^{C}\left(C_{0}\right)}$$

(Equation 3) $$F^{T}\left(T_{t}\right)=\beta^{T}{T_{t}}^{2}+\gamma^{T}T_{t}+\alpha^{T}$$

*F*^*T*^ denotes the equation representing the variations in yield for each crop at a certain period, influenced by the local growing season temperature.

(Equation 4) $$F^{C}\left(C_{t}\right)=\left\{ \begin{array}{ll} \beta^{C}{C_{t}}^{2}+\gamma^{C}C_{t}+\alpha^{C} & \left(C_{t}<700\right)\\ 1.174 & \left(C_{t}\geq 700\right) \end{array}\right.$$

*F*^*C*^ refers to the equation representing the changes in per-acre yield for each crop, influenced by the concentration of carbon dioxide. As carbon dioxide is a well-mixed greenhouse gas, it does not need to be scaled down like surface temperature. Regarding the yield-CO2 concentration function, when the carbon dioxide concentration exceeds or equals 700 ppm, the fertilizer intensity will no longer increase. More detail information can be found in Xu et al.^2^

#### Estimate of human-nature interactions

We calculate the difference between the coupled simulation and the uncoupled simulation (original GCAM scenarios) as the contribution of human-nature interactions, represented by the following formula:

(Equation 5) $$X_{interaction}=X_{coupled}-X_{uncoupled}$$

Here, X represents the various variables of interest in this study, such as the areas of various land uses, food production, and carbon prices. The subscript ‘interaction’ denotes the contribution of human-nature interactions, while ‘coupled’ and ‘uncoupled’ represent the model outputs of the coupled and uncoupled simulations, respectively.

To assess the contribution of human-nature interactions, we conduct the uncoupled simulation to obtain reference values for the variables of interest in this study. Simultaneously, we obtain the carbon dioxide concentration and global warming under various shared socioeconomic pathways (SSPs) to drive the coupled simulation. Based on the carbon dioxide concentration and global warming obtained from the uncoupled simulation, we utilize the downscaling module and yield module mentioned earlier to simulate the yield under the influence of climate change. This yield is then used to drive changes in land use and climate in the GCAM simulation. This process is repeated until equilibrium is reached, with the criterion that the temperature difference between two iterations is less than 0.05°C. In practice, one iteration achieves equilibrium for the SSP1-2.6 and SSP2-4.5 scenarios, while two iterations are needed for the SSP5-Unconstrained scenario to reach equilibrium (see Figure S7). This iterative process reflects the positive feedback mechanism of human-nature interactions in agricultural activities (Figures 1 and 3).

The iteration is represented as follows:

(Equation 6) $$T_{G}^{n}=f\left(T_{G}^{n-1}\right)$$

f represents the GCAM that incorporates the downscaling and yield modules. The termination criterion is $T_{G}^{n}-T_{G}^{n-1}\leq 0.05{}^{\circ}C$.

#### Enhanced managements

We designed additional simple experiments to test the potential of the adaptive managements to cropland expansion. The reported yield loss mitigation from agricultural management adaptation, as reported in ref.,^21^, is as follows: 13% for maize, 1% for soybean, 5% for wheat, and 7% for rice. Applying these mitigation ratios in our experiments, we aim to examine the influence of enhanced management on field expansion, assuming that the mitigation ratios for other crops will mirror those for wheat, and these ratios will remain consistent across various scenarios.

#### Technological advancements

In GCAM, Agriculture Productivity Growth (APG) is based on FAO's projections. The extent of future technological advancements may vary, either being greater or smaller. To test optimistic scenarios of land use changes, we conducted an additional experiment by increasing APG for each region by 1/3, which represents a surpassing of anticipated technological advancements. The design of these experiments for enhanced managements and technological advancement are also presented in Table 1.

### Quantification and statistical analysis

#### Calculation of human–nature interaction effects

The contribution of human–nature interactions was quantified as the difference between the coupled and uncoupled simulations. For each variable of interest, the interaction effect was calculated as:

(Equation 7) $$X_{interaction}=X_{coupled}-X_{uncoupled}$$

where X denotes the variable of interest, including land-use area, crop harvested area, crop production, land-use-related carbon emissions, atmospheric CO2 concentration, carbon price, or global mean surface temperature (GMST). Xcoupled denotes the output from the coupled simulation in which climate-induced yield changes affect land-use allocation and land-use-related emissions. Xuncoupled denotes the corresponding output from the uncoupled GCAM reference simulation without climate-yield feedbacks. Positive values indicate increases caused by human–nature interactions, whereas negative values indicate decreases caused by human–nature interactions.

#### Calculation of cropland sensitivity to warming

The sensitivity of cropland area to warming was calculated as dS/dT, where S denotes global cropland area and T denotes GMST relative to pre-industrial levels. The sensitivity was estimated from changes in cropland area per unit change in GMST across model years. Only years in which both GMST and atmospheric CO2 concentration increased were used for this calculation, to avoid periods in which stabilized or declining forcing would obscure the relationship between warming and cropland response.

#### Calculation of food self-sufficiency ratios

The staple-food self-sufficiency ratio was calculated as the proportion of regional staple-food consumption supplied by domestic staple-food production. Staple foods include wheat, rice, and maize. For each region, the self-sufficiency ratio was calculated as:

(Equation 8) Self_sufficiencyratio=Regionalstaple_foodproductionRegionalstaple_foodconsumption×100%

Changes in self-sufficiency ratios were calculated as the difference between the coupled and uncoupled simulations. Negative values indicate increased dependence on imported staple-food supply, whereas positive values indicate increased domestic staple-food self-sufficiency.

#### Calculation of land-use and crop-area changes

Changes in land-use area and crop harvested area were calculated as the difference between coupled and uncoupled simulations for the same SSP–RCP scenario, region, land-use type, crop, and model year. Global changes were calculated by summing regional values. Regional contributions to global land-use change were calculated as each region’s share of the global change in the corresponding land-use category. End-of-century changes refer to values in 2100 unless otherwise stated.

#### Figure statistics and uncertainty representation

For line plots, thick lines represent global mean values and thin lines represent basin-level values across the 235 GCAM hydrologic basins. For boxplots, the center line indicates the median, boxes indicate the interquartile range, and whiskers indicate the full range from the minimum to maximum values. For fitted relationships, solid lines indicate fitted regression lines, and shaded bands indicate 95% confidence intervals unless otherwise stated. No hypothesis testing was performed unless otherwise stated.

### Additional resources

No additional resources are associated with this study.
