# Supplementary material for: Widespread land-use change may be caused by adaptation to climate-induced yield reductions
Source: iScience. 2026 Jul 29;29(8):116973. doi: 10.1016/j.isci.2026.116973 (PMC13451921; doi:10.1016/j.isci.2026.116973)
Supplement: Supplementary file 1 [file mmc1.pdf]

iScience, Volume *29*

Supplemental information

Widespread land-use change

may be caused by adaptation

to climate-induced yield reductions

Bo Fu (付博), Yongye Jiang (蒋永烨), Xinhao Suo (索鑫浩), Guolong Chen (陈国龙), Yuqin Lai (赖雨亲), Chenxi Liu (刘晨曦), Yuxin Huang (黄雨昕), and Bengang Li (李本纲)

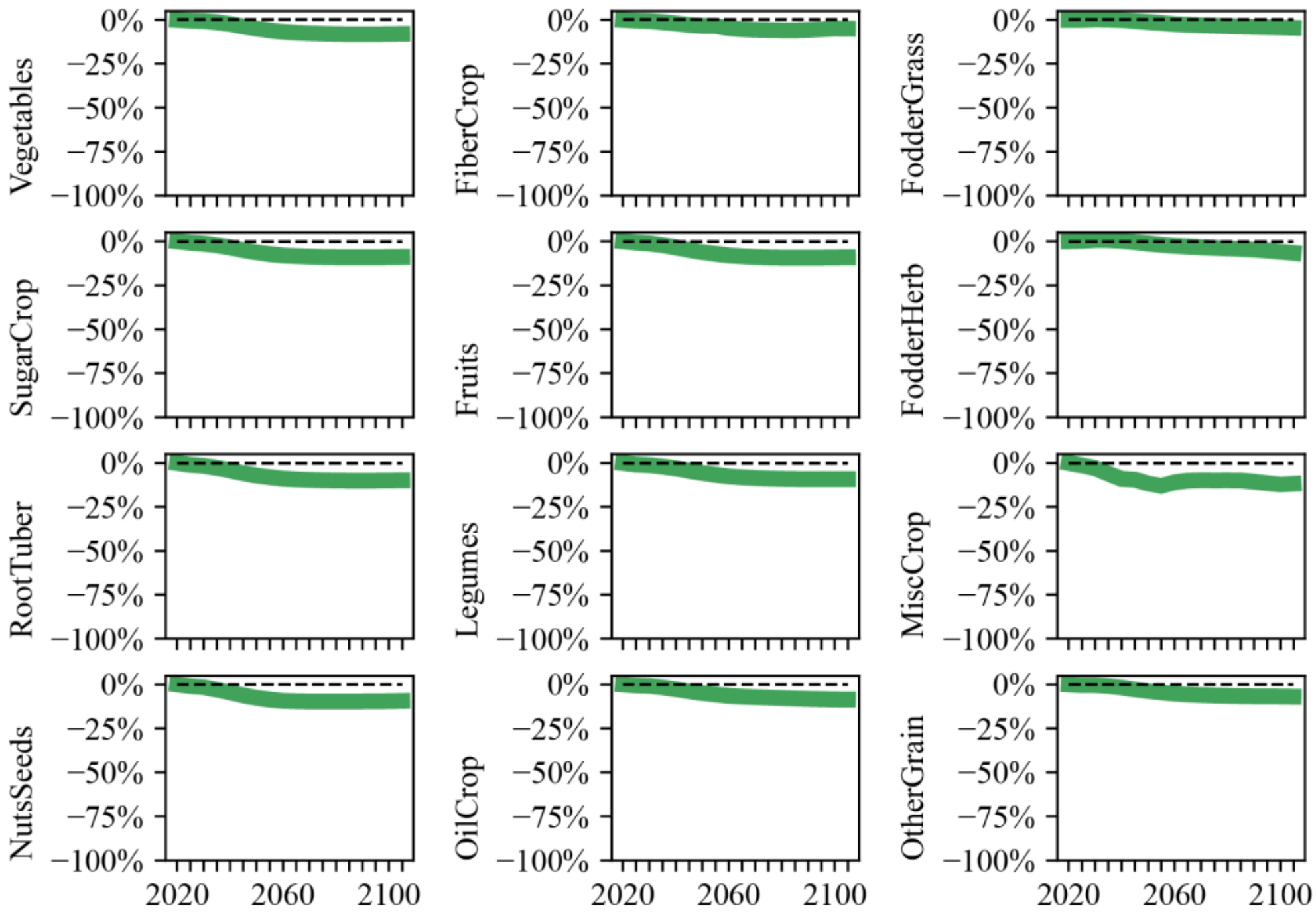

Figure S1. Crop yields affected by climate in the SSP1-2.6 scenario except for the three staple crops (shown in the main text Fig. 1).

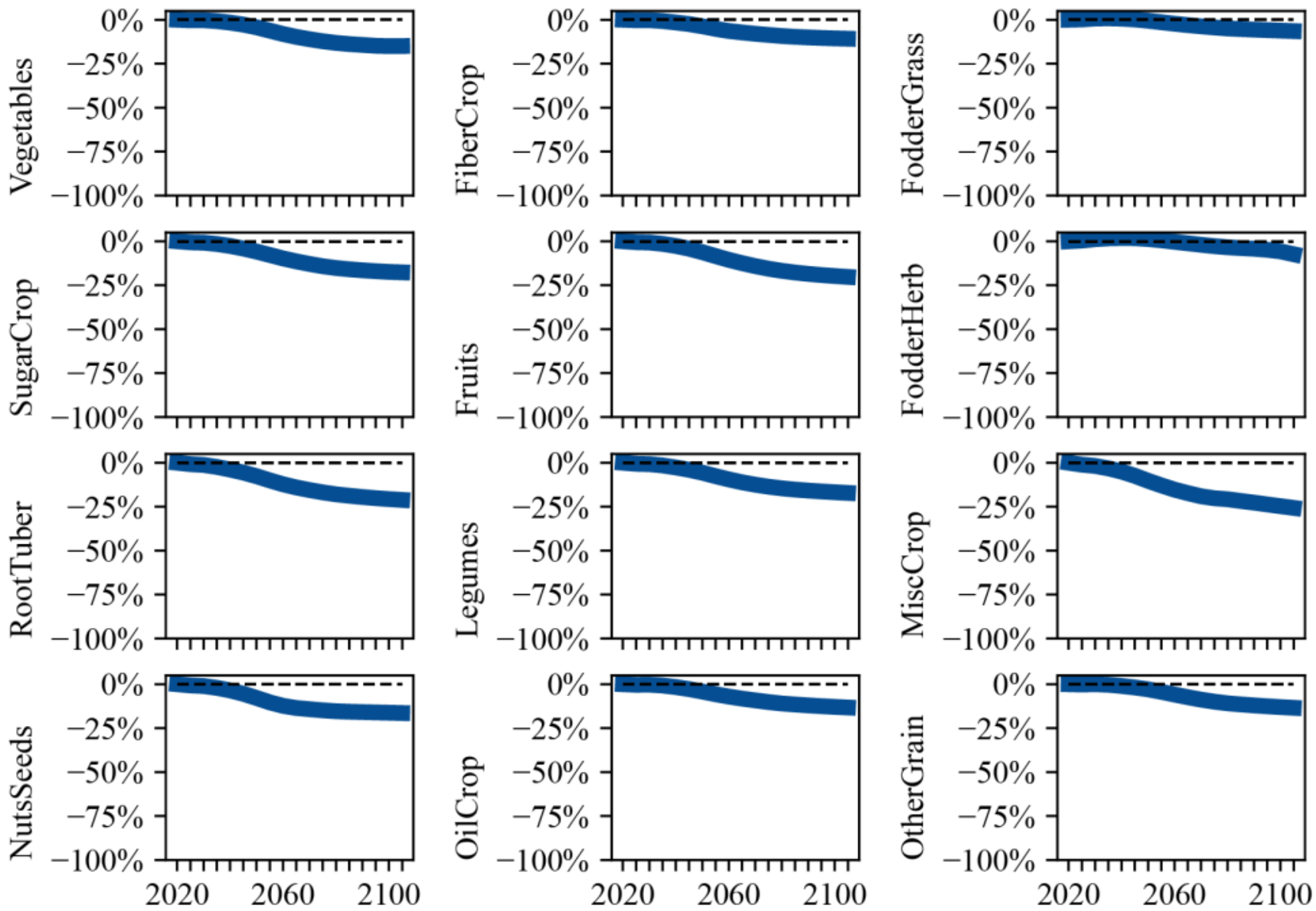

Figure S2. Crop yields affected by climate in the SSP2-4.5 scenario except for the three staple crops (shown in the main text Fig. 1).

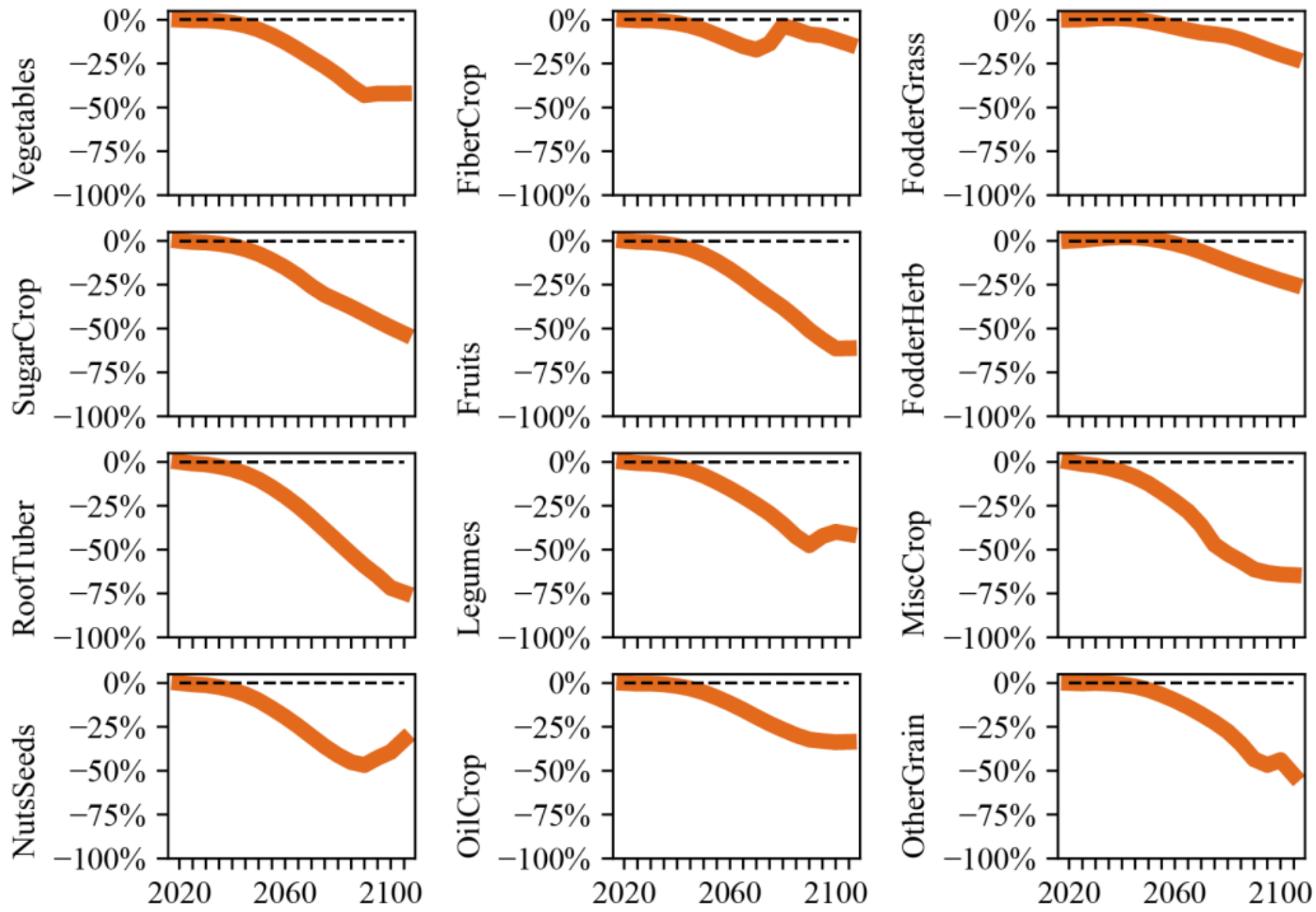

Figure S3. Crop yields affected by climate in the SSP5-Unconstrained scenario except for the three staple crops (shown in the main text Fig. 1).

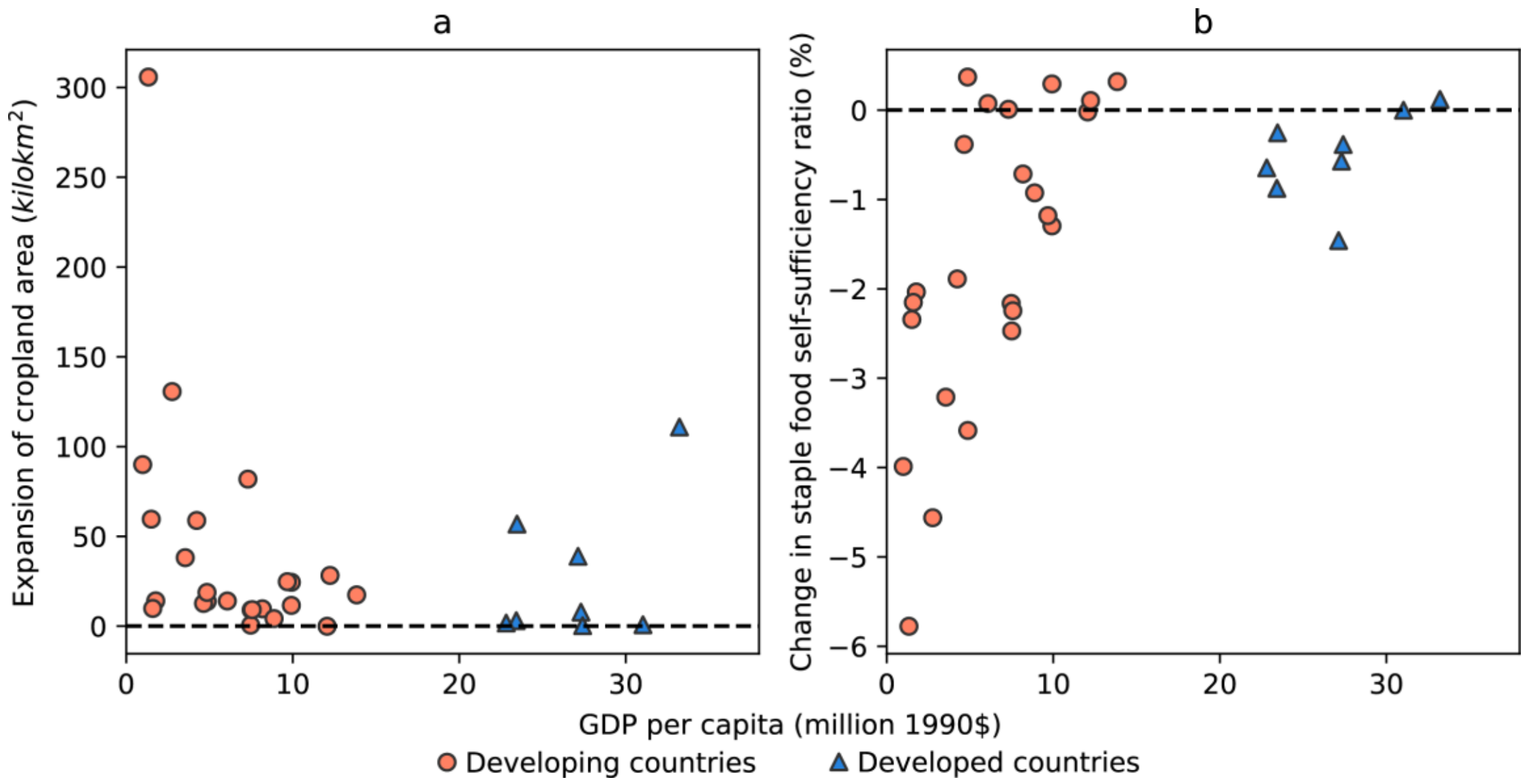

Figure S4. The variance in cropland area and staple food self-sufficiency ratio of countries.

Plots of the expansion of cropland area in 2100 (a) and the change in staple food self-sufficiency ratios (b) against current per capita gross domestic product (GDP) in 2015 for developed (blue) and developing (red) countries.

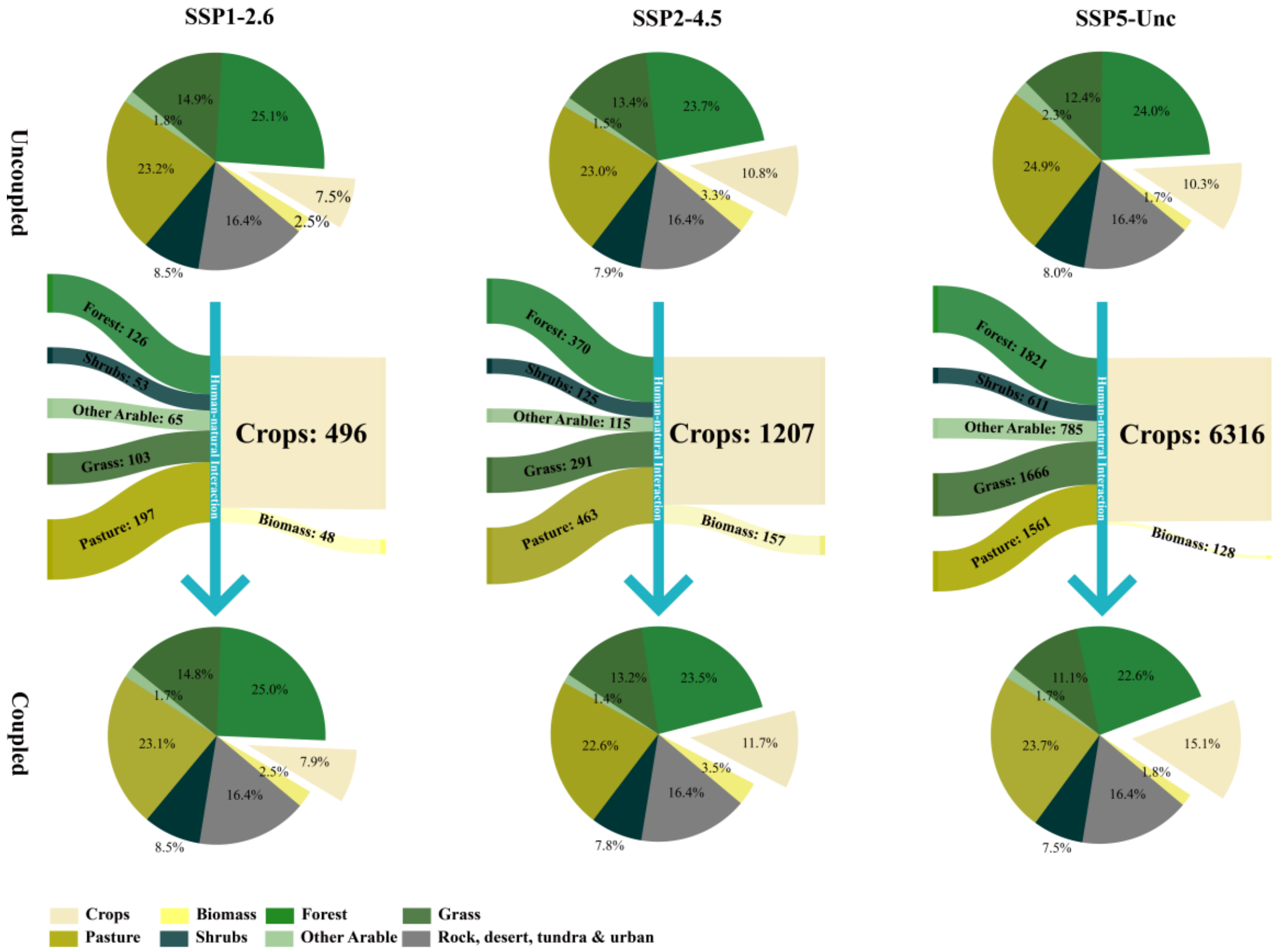

Figure S5. Land use change response to climate-yield effects.

The left-centre-right columns of the figure show the response of land use change to climate-yield effects under the SSP1-2.6, SSP2-4.5, and SSP5-Unconstrained scenarios. The upper pie charts show the share of different land uses at the end of the century without considering climate-yield effects, and the lower pie chart shows the share of different land uses after considering the adaptation of human systems to yield reductions due to climate change. In the middle, the reduction in the area of forests, grasslands, pastures, etc., and the increase in the area of cropland and bioenergy biomass are shown in thousands of square kilometres.

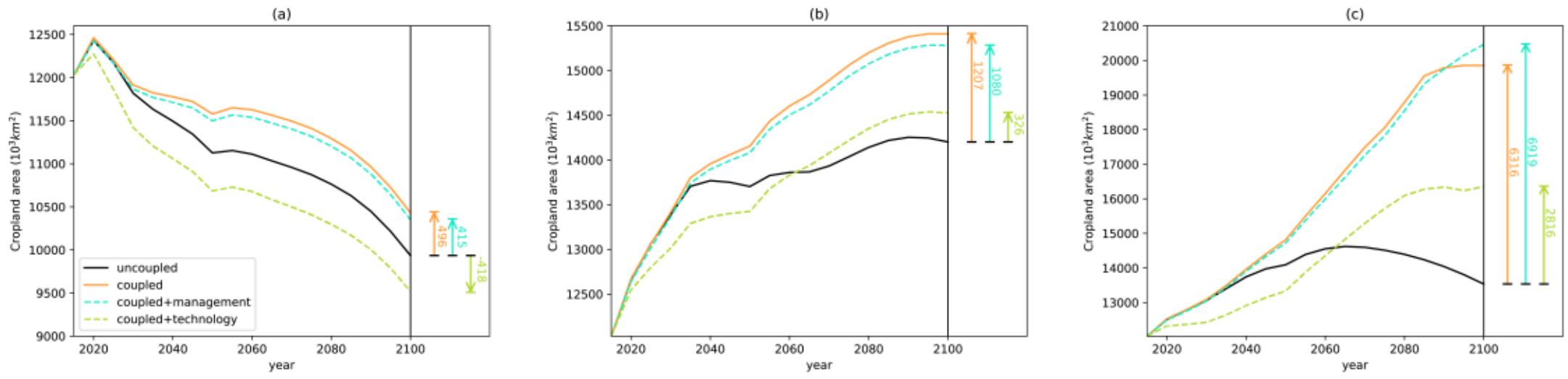

Figure S6. The projected cropland area trends incorporating enhanced management practices and technological advancements.

The figure illustrates the variations in cropland area under diverse climate change scenarios, with (a) representing SSP1-2.6, (b) representing SSP2-4.5, and (c) representing SSP5-Unconstrained. The black lines serve as references, indicating results from uncoupled experiments without climate-yield effects. The orange lines represent the outcomes from coupled experiments. Its deviations from the references are the cropland expansion discussed in the main text. Enhanced managements are denoted by the blue lines, and technological advancements are represented by the green lines in each scenario. The differences from the references are texted on the right side of the figure. This figure shows that, even when considering enhanced managements (blue lines) and technological advancements (green lines), substantial cropland expansion is anticipated, particularly in high-warning scenarios.

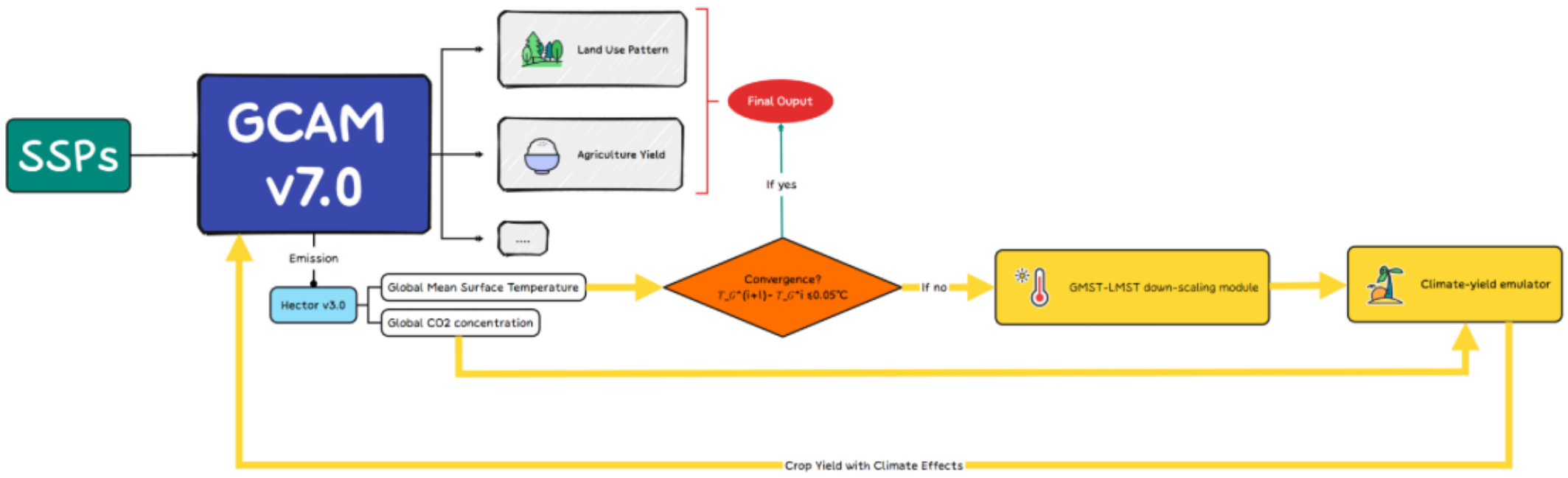

Figure S7. Flowchart of the algorithm for estimating the estimation of human-nature interactions.

SI Table 1. The cropland area expansion, staple food self-sufficiency ratio changes in 2100 in SSP2 scenario, and their GDP per capita in 2015. Region definitions are provided in **SI Table 2**.

Region	Cropland area expansion (kilo km2)	Staple food self-sufficiency ratio changes	GDP per capita in (million 1990$)
Africa_Eastern	90	-4.0%	1.0
Africa_Northern	14	-3.6%	4.9
Africa_Southern	60	-2.3%	1.5
Africa_Western	306	-5.8%	1.3
Argentina	0	0.0%	12.1
Australia_NZ	39	-1.5%	27.1
Brazil	10	-0.7%	8.2
Canada	8	-0.6%	27.3
Central America and Caribbean	13	-0.4%	4.6
Central Asia	19	0.4%	4.9
China	82	0.0%	7.3
Colombia	9	-2.5%	7.5
EU-12	17	0.3%	13.8
EU-15	57	-0.3%	23.5
Europe_Eastern	14	0.1%	6.1
Europe_Non_EU	12	0.3%	9.9
European Free Trade Association	1	0.0%	31.0
India	131	-4.6%	2.8
Indonesia	38	-3.2%	3.5
Japan	3	-0.9%	23.4
Mexico	24	-1.3%	9.9
Middle East	25	-1.2%	9.7

Pakistan	14	-2.0%	1.8
Russia	28	0.1%	12.2
South Africa	1	-2.2%	7.5
South America_Northern	4	-0.9%	8.9
South America_Southern	9	-2.2%	7.6
South Asia	10	-2.2%	1.6
South Korea	2	-0.6%	22.8
Southeast Asia	59	-1.9%	4.2
Taiwan, a Province of People Republic of China	0	-0.4%	27.4
USA	111	0.1%	33.2

SI Table 2. Region definitions in this study, which follows GCAM (https://jgcri.github.io/gcam-doc/overview.html).

GCAM Region	Countries
Africa_Eastern	Burundi, Comoros, Djibouti, Eritrea, Ethiopia, Kenya, Madagascar, Mauritius, Reunion, Rwanda, Sudan, Somalia, Uganda
Africa_Northern	Algeria, Egypt, Western Sahara, Libya, Morocco, Tunisia
Africa_Southern	Angola, Botswana, Lesotho, Mozambique, Malawi, Namibia, Swaziland, Tanzania, Zambia, Zimbabwe
Africa_Western	Benin, Burkina Faso, Central African Republic, Cote d'Ivoire, Cameroon, Democratic Republic of the Congo, Congo, Cape Verde, Gabon, Ghana, Guinea, Gambia, Guinea-Bissau, Equatorial Guinea, Liberia, Mali, Mauritania, Niger, Nigeria, Senegal, Sierra Leone, Sao Tome and Principe, Chad, Togo
Argentina	Argentina
Australia_NZ	Australia, New Zealand
Brazil	Brazil
Canada	Canada
Central America and the Caribbean	Aruba, Anguilla, Netherlands Antilles, Antigua & Barbuda, Bahamas, Belize, Bermuda, Barbados, Costa Rica, Cuba, Cayman Islands, Dominica, Dominican Republic, Guadeloupe, Grenada, Guatemala, Honduras, Haiti, Jamaica, Saint Kitts and Nevis, Saint Lucia, Montserrat, Martinique, Nicaragua, Panama, El Salvador, Trinidad and Tobago, Saint Vincent and the Grenadines
Central Asia	Armenia, Azerbaijan, Georgia, Kazakhstan, Kyrgyzstan, Mongolia, Tajikistan, Turkmenistan, Uzbekistan
China	China
Colombia	Colombia
EU-12	Bulgaria, Cyprus, Czech Republic, Estonia, Hungary, Lithuania, Latvia, Malta, Poland, Romania, Slovakia, Slovenia
EU-15	Andorra, Austria, Belgium, Denmark, Finland, France, Germany, Greece, Greenland, Ireland, Italy, Luxembourg, Monaco, Netherlands, Portugal, Sweden, Spain, United Kingdom

Europe_Eastern	Belarus, Moldova, Ukraine
European Free Trade Association	Iceland, Norway, Switzerland
Europe_Non_EU(ENE)	Albania, Bosnia and Herzegovina, Croatia, Macedonia, Montenegro, Serbia, Turkey
India	India
Indonesia	Indonesia
Japan	Japan
Mexico	Mexico
Middle East	United Arab Emirates, Bahrain, Iran, Iraq, Israel, Jordan, Kuwait, Lebanon, Oman, Palestine, Qatar, Saudi Arabia, Syria, Yemen
Pakistan	Pakistan
Russia	Russia
South Africa	South Africa
South America_Northern	French Guiana, Guyana, Suriname, Venezuela
South America_Southern	Bolivia, Chile, Ecuador, Peru, Paraguay, Uruguay
South Asia	Afghanistan, Bangladesh, Bhutan, Sri Lanka, Maldives, Nepal
Southeast Asia(SE Asia)	American Samoa, Brunei Darussalam, Cocos (Keeling) Islands, Cook Islands, Christmas Island, Fiji, Federated States of Micronesia, Guam, Cambodia, Kiribati, Lao Peoples Democratic Republic, Marshall Islands, Myanmar, Northern Mariana Islands, Malaysia, Mayotte, New Caledonia, Norfolk Island, Niue, Nauru, Pacific Islands Trust Territory, Pitcairn Islands, Philippines, Palau, Papua New Guinea, Democratic Peoples Republic of Korea, French Polynesia, Singapore,

	Solomon Islands, Seychelles, Thailand, Tokelau, Timor Leste, Tonga, Tuvalu, Viet Nam, Vanuatu, Samoa
South Korea	South Korea
Taiwan	Taiwan, a Province of People Republic of China
USA	United States
